# Evaluation of Rheological and Anti-Aging Properties of TPU/Nano-TiO_2_ Composite-Modified Asphalt Binder

**DOI:** 10.3390/ma15093000

**Published:** 2022-04-20

**Authors:** Haidong Ji, Dongpo He, Bo Li, Guanzhong Lu, Chenyu Wang

**Affiliations:** 1 College of Civil Engineering, Northeast Forestry University, Harbin 150000, China; jhd@nefu.edu.cn (H.J.); 384425201@nefu.edu.cn (G.L.); 2017111055@nefu.edu.cn (C.W.); 2 College of Civil Engineering, Lanzhou Jiao Tong University, Lanzhou 730000, China; libolzjtu@hotmail.com

**Keywords:** polyurethane, nano-titanium dioxide, rheological properties, micro-performance, ultraviolet aging

## Abstract

Research on polyurethane-modified asphalt has become very popular. To this end, researchers have explored different ways, such as the use of polyurethane, to improve the road performance of asphalt. However, according to existing experimental research findings, it seems that the use of polyurethane alone cannot completely improve the road performance of asphalt. Therefore, the influence of nano-titanium dioxide and polyurethane on the rheological behavior and anti-ultraviolet aging properties of asphalt was studied. In this research, the rheological and microscopic tests of asphalt were conducted using Dynamic Shear Rheometer, Curved Beam Rheometer, and Fourier Infrared Spectrometer. The results show that the addition of TPU and nano-TiO_2_ to the asphalt not only improves the high- and low-temperature rheological behavior of the asphalt, but also improves the thermal oxygen resistance and UV aging resistance of the asphalt, and prolongs the use performance. Considering economic factors and environmental influences, among all the selected dosages, 4% TPU and 1% nano-TiO_2_ had the best performance.

## 1. Introduction

Asphalt pavement is widely used for expressways because of its advantages of low noise and low dust [1,2]. In the past few decades, pavement diseases (rutting, spalling, and cracking) caused by water damage and traffic load have seriously affected the pavement’s performance and service life, which also has a non-negligible impact on the sustainable development of expressways [3,4,5,6]. At the same time, because the asphalt pavement is exposed to the natural environment throughout the year, there will be complex physical and chemical reactions between the asphalt and the oxygen in the air, ozone, and ultraviolet rays, which will cause damage on the asphalt pavement. In recent years, polyurethane (PU) has been widely used in asphalt pavement engineering due to its excellent mechanical properties, durability, and fatigue resistance [7,8,9]. For example, Jin et al. [10] used polyurethane (PU) and rock asphalt (RA) to prepare a kind of PU composite-modified asphalt with excellent performance at high and low temperatures, which means that PU can make up for the poor low-temperature crack resistance of RA-modified asphalt. Sun et al. [5] not only determined the preparation process of PU-modified asphalt, but also pointed out that PU-modified asphalt and its mixtures have excellent high-temperature stability and water resistance. They also found that the low-temperature crack resistance of PU-modified asphalt mixtures was slightly lower than that of SBS (Styreneic Block Copolymers)-modified asphalt. Jia et al. [11] found that organic montmorillonite (OMMT) can improve the storage performance of thermoplastic polyurethane (TPU)-modified asphalt, and 2% OMMT and 9% TPU were recommended as the optimal dosage. Bzmara et al. [12] found that adding PU to asphalt could improve the low-temperature crack resistance of asphalt, and inferred that PU, as a modifier, could form new chemical bonds after fully reacting with asphalt through the FTIR test. Yao et al. [13] used polyurethane rubber composite-modified asphalt. Through the Marshall test and rutting test, test of polyurethane rubber asphalt mixture’s high-temperature stability, low-temperature crack resistance and water damage resistance, the composite modification advantages were verified.

Studies have found that ultraviolet radiation will cause the aging of asphalt pavements and therefore reduce the service life of the pavement. Moreover, with the change of the global climate, ultraviolet radiation from the sun is becoming more and more intense, which results in the growing impact of ultraviolet aging on the service life of asphalt pavement [14,15]. According to the causes of asphalt aging, aging is divided into thermal-oxidative aging and photooxidative aging. Most researchers mainly focus on the adverse effects of temperature and oxygen on asphalt pavement, but few consider the impact of ultraviolet aging on asphalt pavement [16,17]. However, as an organic material, asphalt and polyurethane are prone to age under the action of temperature, oxygen, water, and ultraviolet radiation [4,17]. Therefore, this article will fully consider the effect of ultraviolet radiation on the macro-rheology and micro-properties of modified asphalt.

At the same time, researchers have carried out a lot of research on nanomaterial-modified asphalt, for example, nano-titanium dioxide (nano-TiO_2_), organic expanded vermiculite (OEVMT), nano-zinc oxide (nano-ZnO), graphene oxide (GO), and carbon nanotubes (CNT) [18,19,20]. Compared with several other nanomaterials, nano-TiO_2_ can not only absorb ultraviolet radiation, but also reflect and scatter ultraviolet radiation. Studies have shown that nano-titanium dioxide has a good shielding effect on ultraviolet rays [21,22].

As shown in previous studies, TPU has been used in conjunction with other materials to try and improve the behavior of asphalt and asphalt mixtures. After adding these modifiers, some asphalt and asphalt mixtures show good mechanical properties. However, so far, no researchers have used titanium dioxide and TPU to improve the performance of asphalt and asphalt mixtures. As a result, in order to continue to improve the literature, in this study, a TPU/TiO_2_-modified asphalt binder was prepared, and the rheological properties and UV aging resistance of the composite-modified asphalt were evaluated.

## 2. Materials and Methods

### 2.1. Materials

#### 2.1.1. Base Asphalt

The asphalt used in this test is 90# base asphalt, whose performance meets the requirements of the Technical Specification for Highway Asphalt Pavement Construction (JTGF40-2019) [23]. The test results are shown in Table 1.

#### 2.1.2. Thermoplastic Polyurethane Elastomer (TPU)

TPU can be divided into the polyester type and the polyether type. The polyether-type polyurethane has good hydrolysis resistance and excellent low-temperature performance because it has an ether bond and low cohesive energy. In this study, polyether-type TPU, provided by Feng Hua Xi Kou Jiu yuan New Materials Co., Ltd., Ningbo City, China, was used, and its performance index is shown in Table 2.

#### 2.1.3. Nano-TiO_2_

Nano-TiO_2_, provided by Guangzhou Nano Chemical Technology Co., Ltd., (Guangzhou, China) was used in this study, and its basic performance indexes are shown in Table 3.

### 2.2. Test Methods

#### 2.2.1. Preparation Method of Nano-TiO_2_/TPU Composite-Modified Asphalt

**Figure 1:**
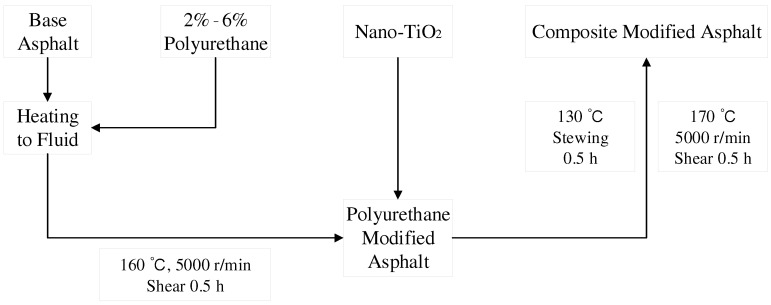
The preparation process of modified asphalt.

In the process of preparing composite-modified asphalt, the performance of composite materials is closely related to the preparation process. Refer to previous reports [10,24] using the high-speed shear physical fusion method to prepare TPU/nano-TiO_2_ composite-modified asphalt. The specific preparation method is as follows: Preheat the base pitch to 160 °C and stir at a constant speed to prevent it from generating bubbles. Add different amounts of TPU (2%, 4%, 6%) to the base asphalt and mix it evenly with a SGJ100 mixer. Make asphalt and TPU powder fully compatible. Then, use the JRJ-300-I shearing machine to cut for 30 min at 5000 r/min. Then, at 170 °C, 1%, 2%, and 3% (by weight of base asphalt) nano-TiO_2_ is added to the asphalt several times in small amounts. After using a mixer to quickly disperse it evenly, use the shearing machine again to continue shearing for 30 min at a speed of 5000 r/min. The prepared asphalt was maintained in an oven at 130 °C for 0.5 h to obtain a TPU/nano-TiO_2_-modified asphalt. The preparation process is shown in Figure 1. To avoid errors caused by thermal-oxidative aging during the preparation of the modified asphalt, the base asphalt of the control group also needs to go through the same operation process described above. For the convenience of the following description, for example, base asphalt is denoted as BA and 2TPU-1nanoTiO_2_ as 2/1.

#### 2.2.2. Ultraviolet Aging Test Method

**Figure 2:**
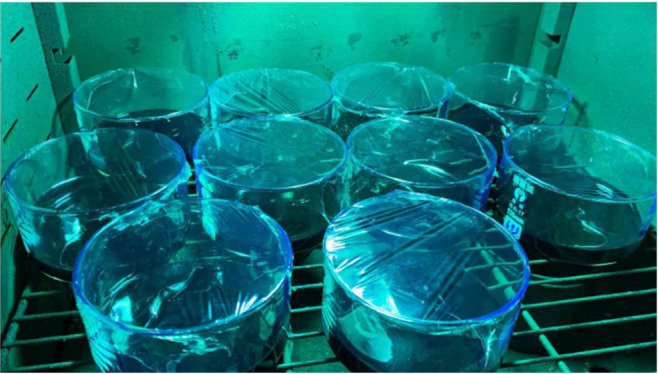
Self-made UV aging oven.

The aging method of RTFOT (Rolling Thin-Film Oven Test) was carried out according to the specification: The Test Specification for Asphalt and Asphalt Mixture of Highway Engineering (JTG E20-2011) [25]. The UV aging test was carried out with a self-made UV aging oven, as shown in Figure 2. The steps of the UV aging test are as follows: Firstly, the asphalt sample aged by RTFOT is poured into a 150 mm-diameter transparent glass culture dish, and the thickness of the asphalt sample is about 0.5 mm. Then, the asphalt sample is placed into a self-made UV aging oven with continuous light for 15 days to make the asphalt sample fully aged. The light intensity in the oven is 420 W/m^2^ and the temperature is 64 °C; by calculation, it is equivalent to the amount of ultraviolet radiation in China’s Heilongjiang Province for one year [26].

### 2.3. Experiment Method

#### 2.3.1. DSR Test

According to AASHTO T315, this paper used the temperature scanning mode of the MCR302 Dynamic Shear Rheometer (DSR) to test the modified asphalt. The complex modulus, phase angle, and rutting factor were used as the high-temperature performance indicators of modified asphalt. Among them, the loading frequency was 10 rad/s, the strain was 12%, and the test temperatures were 43, 49, 55, 61, 67, 73, 79, and 85 °C.

#### 2.3.2. BBR Test

This study was conducted according to ASTM D6648 and AASHTO T313. The TE-BBR low-temperature bending rheometer (BBR) was used to measure the creep stiffness modulus, S, and creep rate, m, of the samples at different temperatures. Use S/m to evaluate the low-temperature crack resistance of modified asphalt. The test temperatures were −12, −18, −24, and −30 °C.

#### 2.3.3. FTIR Test

In order to analyze the modification mechanism of the modified asphalt, this experiment used the Spectrum 400 Fourier transform infrared spectrometer to obtain the infrared spectra before and after asphalt modification. The resolution was 4 cm^−1^, the number of scans was 32, and the test range was 450–4000 cm^−1^.

## 3. Results

### 3.1. High-Temperature Performance

#### 3.1.1. Complex Modulus, G*, and Phase Angle, δ

**Figure 3:**
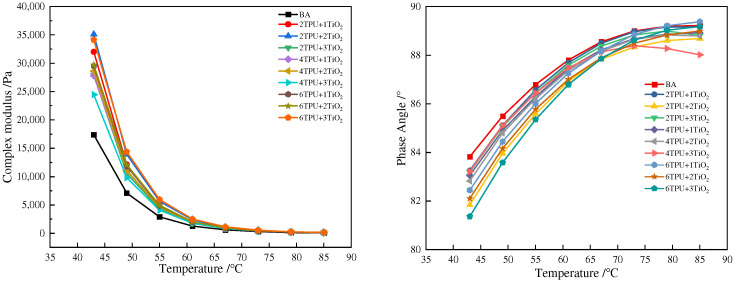
The curve of G* and *δ* with temperature.

The complex modulus and phase angle are indispensable indicators to describe the rheological properties of asphalt at high temperatures. The complex modulus is directly proportional to the high-temperature resistance of asphalt. Figure 3 presents a graph showing the changes of base asphalt and modified asphalt with temperature.

It can be seen from Figure 3 that as the temperature increases, the complex modulus of asphalt gradually decreases, and the phase angle gradually increases. This shows that with the increase of temperature, the high-temperature stability of asphalt will become worse. In Figure 3, the temperature increases from 43 to 85 °C, and the phase angle of the pitch increases sequentially by 5.4°, 5.9°, 6.8°, 5.6°, 5.9°, 6.0°, 4.8°, 6.9°, 6.9°, and 7.8°. Although the growth rate of the phase angle of modified asphalt is greater than that of base asphalt, it can be seen from Figure 3 that in the range of 43–67 °C, the phase angle, δ, of the modified asphalt was smaller than the phase angle, δ, of the base asphalt. In this temperature range, the modified asphalt has better resistance to deformation under load.

#### 3.1.2. Rutting Factor, G*/Sinδ

**Figure 4:**
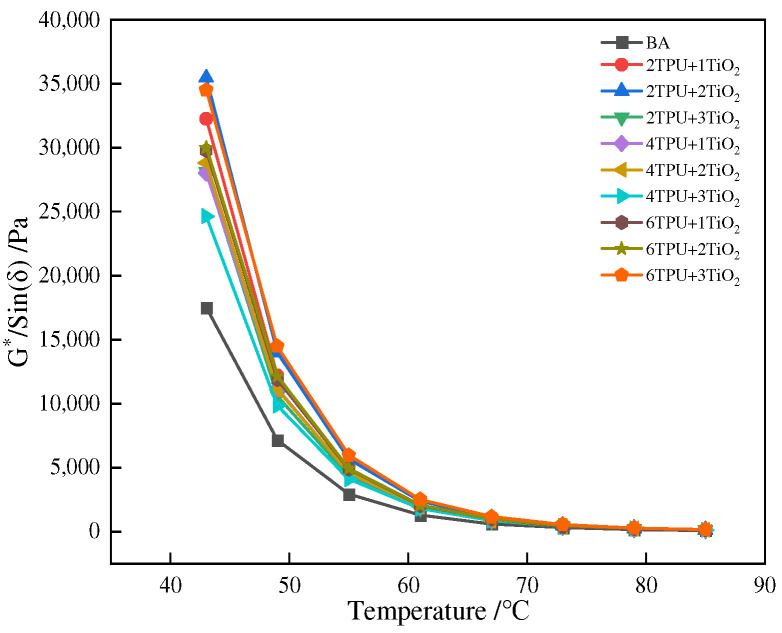
The curve of G*/Sinδ with temperature.

The rutting factor is an index to evaluate the ability of asphalt to resist high-temperature rutting deformation. The larger the rutting factor, the stronger the asphalt’s ability to resist high-temperature rutting deformation. Figure 4 is a graph of asphalt rutting factor versus temperature.

It can be seen from Figure 4 that the rutting factor gradually decreases with increasing temperature. In the range of 43~80 °C, the rutting factor of modified asphalt was greater than that of base asphalt. This indicates that the incorporation of TPU and nano-TiO_2_ improved the rutting factor of asphalt. Composite-modified asphalt has good resistance to rutting deformation at high temperatures. It has good high-temperature stability under high-temperature conditions. It can also be seen from Figure 4 that when the content of TPU is the same and the content of nano-TiO_2_ is 1–2%, the rutting factor shows an upward trend. When the content of nano-TiO_2_ is 2–3%, the rutting factor shows a downward trend. However, when the content of TPU is a single variable, the change of rutting factor appears to be irregular. This means that in the process of TPU nano-TiO_2_ synergistically improving the anti-rutting ability of asphalt at high temperatures, nano-TiO_2_ plays a leading role.

### 3.2. Low-Temperature Performance

**Figure 5:**
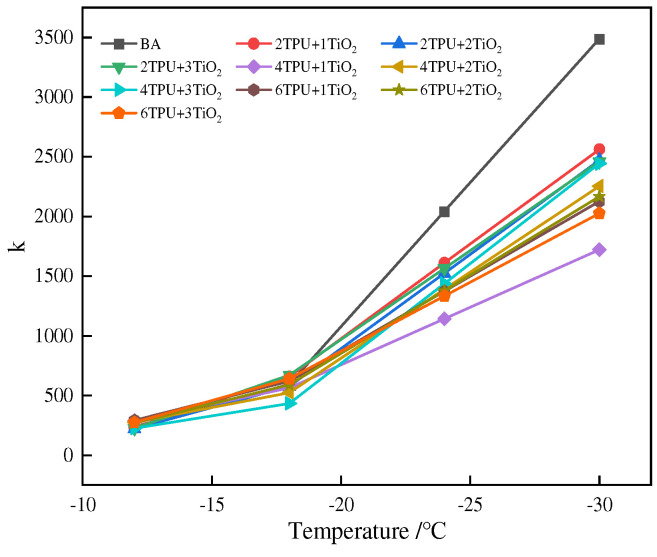
The curve of k with temperature.

In order to simply and directly evaluate the low-temperature crack resistance of modified asphalt, this study used S/m for comparative analysis. S/m is inversely proportional to the low-temperature crack resistance of asphalt. Figure 5 shows the curve of S/m versus temperature for asphalt at different temperatures.

It can be seen from Figure 5 that in the range of −12 to 30 °C, the S/m of the modified asphalt was much smaller than that of the base asphalt. This shows that TPU and nano-TiO_2_ can improve the ability of asphalt to resist low-temperature cracking. The S/m of composite-modified asphalt with 4% TPU and 1% nano-TiO_2_ is the smallest. This shows that the low-temperature crack resistance of the modified asphalt was the best. In Figure 5, when the amount of nano-TiO_2_ is constant, with the increase of the amount of TPU, S/m shows a trend of first decreasing and then increasing. This shows that TPU can improve the low-temperature performance of asphalt. As a result, the low-temperature performance of the modified asphalt is reduced. However, when the content of TPU is constant, as the content of nano-TiO_2_ increases, the low-temperature crack resistance of asphalt seems irregular. This means that TPU plays a leading role in improving the low-temperature crack resistance of asphalt. It can also be seen from Figure 5 that in the range of −12 to −18 °C, S/m slowly increased, while in the range of −30 to −18 °C, S/m sharply decreased. This is caused by the increase in the energy of the molecular movement inside the asphalt due to the increase in temperature, and the molecular structure is in an unstable state. Before −18 °C, the S/m value of base asphalt and modified asphalt was not much different, but after −18 °C, the S/m value of modified asphalt was much smaller than that of base asphalt. This shows that the composite-modified asphalt can better reflect its superiority in low-temperature crack resistance after −18 °C.

### 3.3. An Evaluation of the Anti-Aging Performance of Asphalt Based on CMI of DSR

In this paper, the complex modulus aging index (CMI) was used to evaluate the anti-aging performance of the sample asphalt [27]. The formula is shown in Equation (1):

(1) $$\mathrm{CMI}=\frac{G_{after\;aging}^{*}}{G_{before\;aging}^{*}}$$

where G^*^_after aging_ is the composite modulus of asphalt after aging, and G^*^_before aging_ is the composite modulus of asphalt before aging.

**Figure 6:**
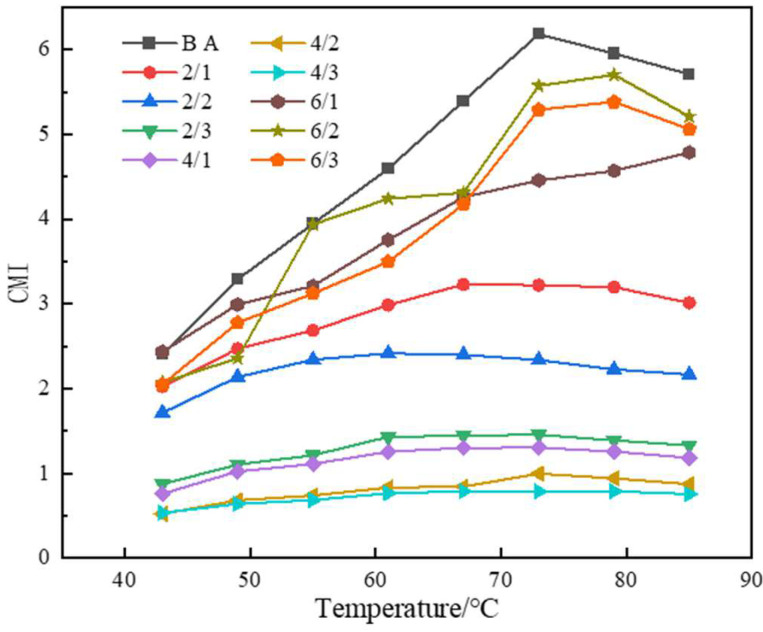
Temperature variation curves of different asphalt CMI.

Figure 6 shows the CMI values of different asphalts after aging. It can be seen that after modification with TPU and nano-TiO_2_, the anti-ultraviolet aging ability of asphalt was significantly improved.

In Figure 6, by comparing the CMI values of 10 asphalts, it was found that the CMI of the modified asphalt was smaller than that of the base asphalt, which indicates that the modified asphalt has good UV aging resistance. However, too much polyurethane will also be degraded during the aging process, which eventually leads to the highest CMI values of 6/1, 6/2, and 6/3 in the modified asphalt.

To determine the change of TPU content during the aging process, infrared spectroscopy tests were carried out on nine modified asphalts. As TPU undergoes thermal oxidative aging and ultraviolet aging, the N-C bond (in the infrared spectrum, the absorption peak of its bending vibration is at 1690–1590 cm^−1^) is destroyed to form amino radicals and so on. Therefore, this article used the change of N-C content before and after aging (*M_N__-C_*) to evaluate the degradation degree of TPU in asphalt. The calculation formula of *M_N-C_* is shown in Equation (2):

(2) $$M_{N\text{-}C}=\frac{A_{0}-A_{t}}{A_{0}}$$

where *A*_0_ is the peak area at 1690–1590 cm^−1^ before asphalt aging, and *A_t_* is the peak area at 1690–1590 cm^−1^ after asphalt aging.

**Figure 7:**
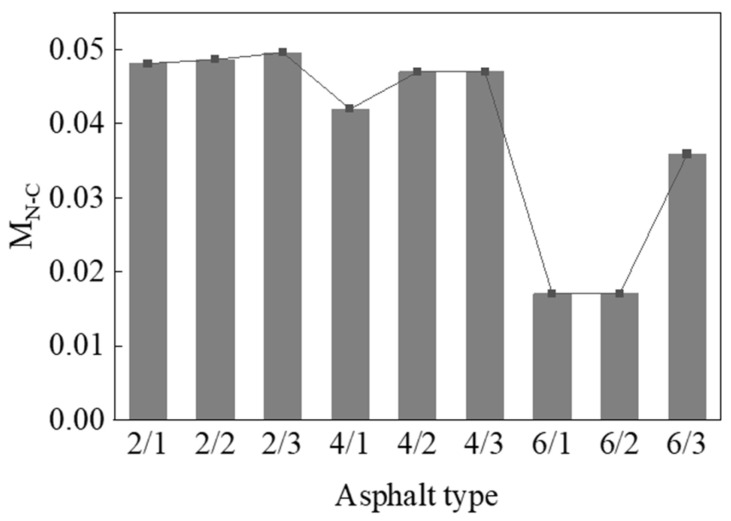
Changes of N-C bond content before and after asphalt aging.

Figure 7 shows the change in N-C bond content of modified asphalt before and after aging. It can be seen from Figure 7 that the N-C bond content of modified asphalt with 2% TPU and 4% modified asphalt was approximately the same, while the N-C bond content of modified asphalt with TPU added at 6% was significantly reduced. This means that during the aging process, the excess TPU is degraded. When the content of TPU was the same, as the amount of nano-TiO_2_ added increased, the N-C bond content of the modified asphalt increased, which shows that nano-TiO_2_ can effectively inhibit the degradation of polyurethane. It can be determined that nano-TiO_2_ can effectively improve the anti-ultraviolet aging ability of polyurethane-modified asphalt. This is mainly due to the fact that when nano-TiO_2_ is irradiated by ultraviolet radiation, when the photon energy is greater than the bandgap energy of TiO_2_, the electrons in the valence band are excited to cross the forbidden band and reach the conduction band. At the same time, corresponding holes are generated, thereby forming hole–electron pairs. In the process of photo-generated electrons and photo-generated holes, it has strong oxidizing ability and reducing ability, and a redox reaction occurs with its surface system attachment and oxygen, respectively, to generate superoxide anion, $O_{2}^{-}$, and hydroxyl radical, OH^−^. Therefore, most organic pollutants can be oxidized by it. Thereby, the degradation of polyurethane is inhibited, the effect of organic pollutants and asphalt is reduced, and the UV aging resistance of the polyurethane-modified asphalt is improved. 

### 3.4. Modification Mechanism and Anti-Ultraviolet Aging Mechanism

#### 3.4.1. Modification Mechanism Analysis

**Figure 8:**
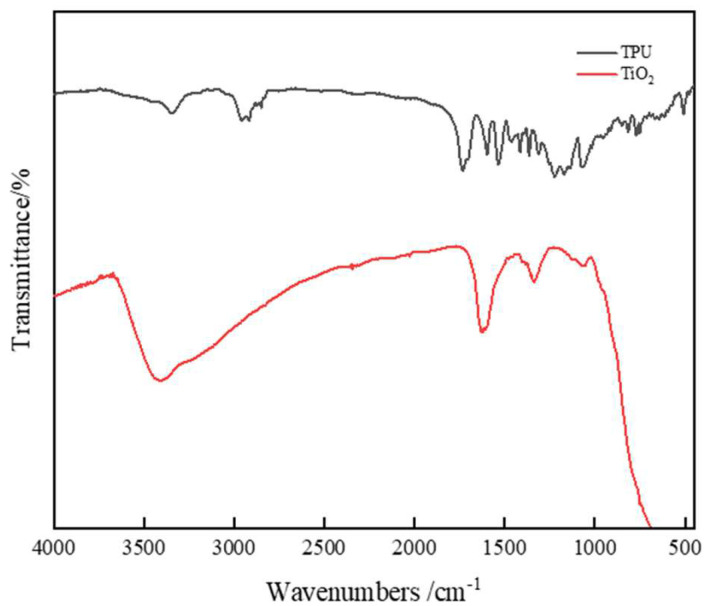
FTIR diagram of TPU and nano-TiO_2_.

**Figure 9:**
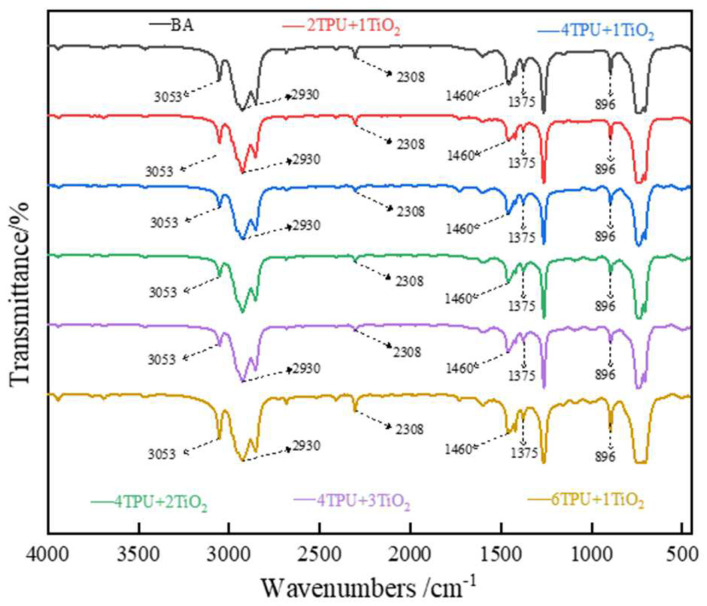
FTIR diagram of asphalt binder.

The above experiments show that TPU/nano-TiO_2_ composite-modified asphalt has good high- and low-temperature rheological properties. However, we do not know the mechanism of action of TPU and nano-TiO_2_ in base asphalt. Therefore, to determine the mechanism of action of TPU and nano-TiO_2_ in asphalt, several modified asphalts (BA, 2/1, 4/1, 4/2, 4/3, 6/1) were selected for FTIR testing. Figure 8 presents the FTIR diagram of TPU and nano-TiO_2_ and Figure 9 shows the FTIR diagram of the asphalt before and after modification.

It can be seen from Figure 9 that the modified asphalt has some new characteristic peaks and the original characteristic peaks increased or decreased. This indicates that the base asphalt may have a chemical reaction with some molecules in TPU or nano-TiO_2_, which leads to changes in functional groups. In Figure 9, a more obvious absorption peak appeared at the wavenumber of the modified asphalt at 1730 cm^−1^, which is mainly caused by the vibration of the urethane group, urea group, and carbon group (C=O) in the amide group [28,29]. Peaks at 3053, 2930, 2308, 1460, and 896 cm^−1^ are caused by =C-H stretching vibration, CH_2_ antisymmetric stretching vibration, C-H in-plane bending vibration, and C-H(=C-H) out-of-plane bending vibration in the unsaturated benzene ring, respectively. Two strong absorption peaks appeared at 1460 and 1375 cm^−1^, which are caused by the addition reaction between the isocyanate in the polyurethane and the aromatic compound in the base asphalt. It can also be seen from Figure 9 that the peak area corresponding to the above-mentioned wavenumbers corresponding to the four doping levels of 2/1, 4/1, 4/2, and 4/3 has been reduced by 34%, 61%, 60%, and 29%, respectively. However, only the peak area of 6/1 increased by 6%. This is because the polyurethane macromolecular segments in 2/1, 4/1, 4/2, and 4/3 react chemically with the base pitch to form new functional groups. Therefore, some peak areas will decrease. However, the polyurethane in the 6/1-modified asphalt will agglomerate, and only a small amount of polyurethane will react with the base asphalt. Therefore, new functional groups will appear in the mixed modified asphalt, and the corresponding peak area is also larger than that of the base asphalt.

From Figure 8 and Figure 9 as a whole, in addition to the new functional groups generated by TPU and matrix pitch, other redundant peaks are inherent to nano-TiO_2_ itself. Since the experiment requires very few samples, and random sampling can detect the presence of nano-TiO_2_ particles by FTIR, this shows that nano-TiO_2_ is evenly distributed in the composite modified asphalt material, and nano-TiO_2_ exists only between TPU and matrix asphalt molecules in a physical bridging manner, making the network structure more stable.

#### 3.4.2. Anti-Ultraviolet Aging Mechanism

**Figure 10:**
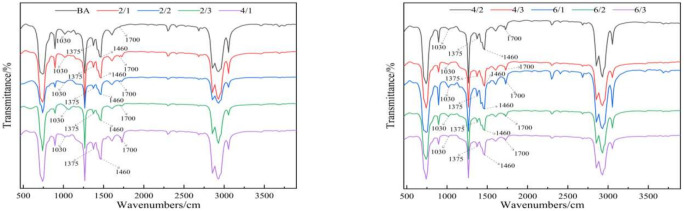
An infrared spectrum of asphalt before aging.

**Figure 11:**
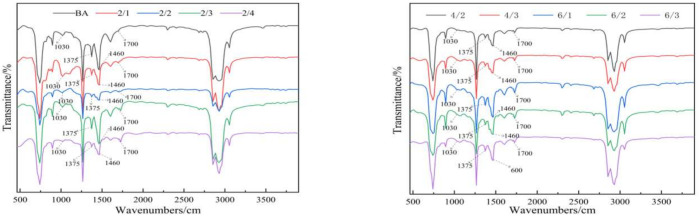
Infrared spectrum of asphalt after aging.

In the process of thermal oxidation aging and photooxidation aging, asphalt will undergo an oxidation reaction, which can generate components containing carbonyl and sulfoxide groups such as aldehydes, ketones, esters, or carboxylic acids. The infrared spectrum of asphalt samples before and after aging is shown in Figure 10 and Figure 11.

In this paper, the absorption peak areas of carbonyl and sulfoxide groups in the infrared spectra of all asphalt before and after aging were integrated, and *CI* and *SI* were calculated as the evaluation indexes of the aging degree [30,31]. The calculation formulas are shown in Equations (3) and (4):

(3) $$CI=\frac{A_{c=o}}{A_{C\text{-}H}}$$

(4) $$SI=\frac{A_{S=O}}{A_{C\text{-}H}}$$

where *A_C=O_* is the absorption peak area of carbonyl, *A_S=O_* is the absorption peak area of the sulfoxide group, and *A_C-H_* is the absorption peak area of CH_3_.

To avoid the error caused by the initial value drift of *CI* and *SI*, the paper also used the normalized carbonyl index (*NSI*) and the normalized sulfoxide index (*NSI*) to compare the decay rate of asphalt aging [32]. The specific calculation formulas are shown in Equations (5) and (6):

(5) $$NCI=\frac{CI_{t}-CI_{0}}{CI_{0}}$$

(6) $$NSI=\frac{SI_{t}-SI_{0}}{SI_{0}}$$

where *CI*_0_ and *CI_t_* represent the carbonyl index before and after aging, and *SI*_0_ and *SI_t_* represent the sulfoxide index before and after aging.

**Figure 12:**
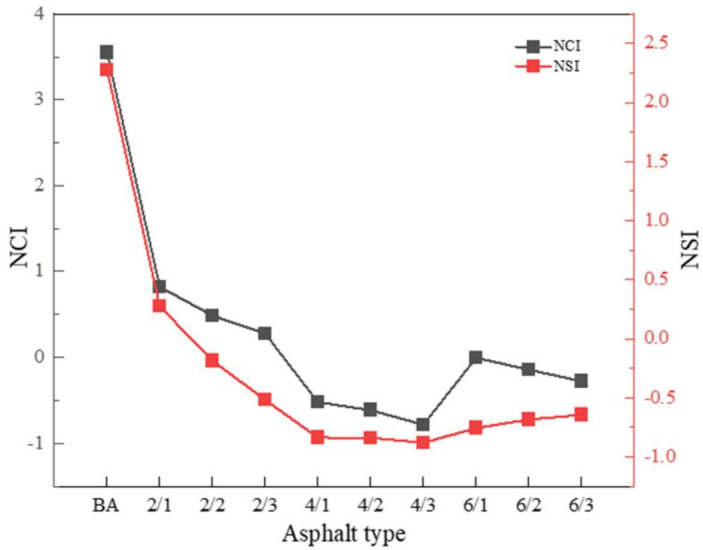
Normalized carbonyl index (*NCI*) and normalized sulfoxide index (*NSI*) of different asphalts.

To evaluate the effect of nano-TiO_2_ on the aging of polyurethane-modified asphalt, the *NCI* and *NSI* of the aging asphalt were calculated, and the results are shown in Figure 12.

In Figure 12, it can be clearly seen that the *NCI* and *NSI* values of the base asphalt were the largest, and with the addition of nano-TiO_2_, the *NCI* and *NSI* values were continuously decreasing.

This shows that nano-TiO_2_ can effectively improve the thermal oxygen and ultraviolet aging resistance of the polyurethane-modified asphalt. When the amount of TPU added was increased from 2% to 4%, *NCI* and *NSI* significantly decreased, decreasing by 163%, 226%, and 382%, and 407%, 355%, and 70%, respectively. However, when the amount of TPU added was increased from 4% to 6%, *NCI* and *NSI* increased by 99%, 82%, and 65%, and 9%, 22%, and 27%, respectively. This explains that if the amount of TPU added is within a certain range, TPU can improve the thermal oxidation resistance and UV aging resistance of asphalt, but when too much TPU is added, TPU will be degraded by aging, resulting in poor asphalt performance.

## 4. Conclusions

In this work, a composite-modified asphalt binder was prepared using TPU and nano-TiO_2_. The high-temperature anti-rutting performance, low-temperature anti-cracking performance, and anti-aging ability of the composite-modified asphalt binder were studied. The effects of TPU and nano-TiO_2_ on the physical, chemical, and microscopic properties of the composite-modified asphalt binders were evaluated. The following general results are the main outputs of this comprehensive study:

(1) TPU and nano-TiO_2_ can have a very good synergistic effect on asphalt, and each exerts its performance advantages. Nano-TiO_2_ plays a leading role in improving the high-temperature stability of asphalt binders. However, in the process of improving the low-temperature crack resistance of asphalt binders, TPU plays a leading role.
(2) Adding an appropriate amount of nano-TiO_2_ to asphalt can inhibit the degradation of TPU, and improve the UV aging resistance of modified asphalt by inhibiting the formation of carbonyl and sulfoxide groups. Therefore, when the TPU content is 4% and the nano-titanium dioxide content is 1%, the use efficiency of asphalt is the highest.

## Figures and Tables

**Table 1:** Basic physical properties of base asphalt.

Technical Index	Test Value	Requirements of The Specification
25 °C penetration/0.1 mm Softening point/°C	80.5 47	80–100 ≥44
5 °C ductility/cm	7.8	
Flashpoint/°C	322	≥245

**Table 2:** The basic performance index of TPU.

Technical Index	Test Data	Test Method (ASTM)
Density/(g/cm³)	1.13	D792
Hardness/ShoreA	95	D2240
Tensile strength/(Mpa)	35	D412
Elongation/%	600	D412
Tear strength/(kg/cm)	130	D0624

**Table 3:** Basic performance indexes of nano-TiO_2_.

Performance Index	Particle Size	Purity	Density	Crystal Form
Test data	5 nm	99.9%	3.9 g/cm³	anatase

## Data Availability

The data presented in this study are available upon request of the first author.
